# Frequency-Dependent Interictal Neuromagnetic Activities in Children With Benign Epilepsy With Centrotemporal Spikes: A Magnetoencephalography (MEG) Study

**DOI:** 10.3389/fnhum.2020.00264

**Published:** 2020-07-10

**Authors:** Tingting Zhang, Qi Shi, Yihan Li, Yuan Gao, Jintao Sun, Ailiang Miao, Caiyun Wu, Qiqi Chen, Zheng Hu, Hu Guo, Xiaoshan Wang

**Affiliations:** ^1^Department of Neurology, Nanjing Brain Hospital, Nanjing Medical University, Nanjing, China; ^2^MEG Center, Nanjing Brain Hospital, Nanjing, China; ^3^Department of Neurology, Nanjing Children’s Hospital, Nanjing, China

**Keywords:** benign epilepsy with centrotemporal spikes, magnetoencephalography, magnetic source imaging, interictal epileptiform discharges, low- to high-frequency bands

## Abstract

**Objective**: This study aimed to investigate interictal neuromagnetic activities in the low- to high-frequency ranges in patients with benign epilepsy with centrotemporal spikes (BECTS), especially those without interictal epileptiform discharges (IEDs).

**Methods**: We studied 21 clinically-diagnosed BECTS patients and 11 age-matched healthy controls (HC) using high-sampling magnetoencephalography (MEG). Neuromagnetic sources were assessed with accumulated source imaging (ASI). The MEG data were analyzed in seven frequency bands. The MEG recordings distinguished BECTS without IEDs (*n* = 10) from those with IEDs (*n* = 11) and HC (*n* = 11).

**Results**: At 1–4 Hz, the neuromagnetic activities in healthy subjects tended to locate at the precuneus/posterior cingulate, while those of the BECTS patients without IEDs tended to locate at the medial frontal cortex (MFC) compared to BECTS patients with IEDs. The MEG source imaging at 30–80 Hz revealed that BECTS patients without IEDs had higher occurrences of interictal brain activity in the medial temporal lobe (MTL) compared to controls and the brain activity strength seemed to be weaker. There was a significant correlation between the source strength of the interictal gamma oscillations of BECTS patients without IEDs and the duration of epilepsy.

**Conclusions**: IEDs might disrupt the default mode network (DMN). Aberrant brain activities in BECTS patients without IEDs were associated with cognitive areas of the brain. The strength of gamma oscillations in the chronic epilepsy state reflected the duration of BECTS.

**Significance**: MEG could reveal the aberrant neural activities in BECTS patients during the interictal period, and such abnormality is frequency-dependent. Gamma oscillations could be used to identify BECTS patients without IEDs.

## Introduction

Benign epilepsy with centrotemporal spikes (BECTS, Rolandic epilepsy) is the most common childhood epilepsy syndrome and shows spontaneous remission in adolescence. The age of onset is from one to 14 years old. It occurs predominantly between the ages of seven to ten and is more common in males (Panayiotopoulos et al., 2008; Callenbach et al., 2010). Interictal electroencephalography (EEG) typically shows a normal background with high amplitude centrotemporal spikes (Parisi et al., 2017). Although BECTS patients are believed to have good prognoses, a series of studies have gradually questioned the concept of BECTS as benign (Loiseau and Duché, 1989; Genizi et al., 2012; Ciumas et al., 2017).

Children usually show some degree of brain dysfunction, especially with the presence of interictal epileptiform discharges (IEDs; Ibrahim et al., 2014). IEDs have been proven to disrupt resting-state brain activity (Fahoum et al., 2013). A general linear model (Masterton et al., 2010) and an event-related independent component analysis (Masterton et al., 2013) have shown that IED-related brain activation was in the centrotemporal areas in BECTS patients. The pathogenesis of epilepsy may include both transient and chronic dysfunction. The transient state of epilepsy is accompanied by IED-related blood-oxygen-level-dependent (BOLD) activation in the regions specific to the syndrome while chronic epileptogenic processes may involve stable changes in functional neural circuit organization (Zhu et al., 2015). Hence, even in the patients without IEDs, aberrant brain activity also exhibits (Li et al., 2009; Mankinen et al., 2011), which is not associated with the number of IEDs (Tristano et al., 2018). A recent study (Zhu et al., 2015) based on simultaneous EEG and functional magnetic resonance imaging (fMRI) observed abnormal neural activities during an interictal period in BECTS, which were associated with the presence or absence of IEDs. Both transient abnormalities and chronic abnormalities may be involved in the development and expression of BECTS.

Functional MRI studies, which measures very-low-frequency brain activity by calculating the amplitude of low-frequency fluctuation, have shown alterations in the resting brain activity of BECTS patients in different frequency bands (Tan et al., 2018). The results suggested that brain regions may play a role in specific frequency domains (Zhan et al., 2014). Hence, it is relevant to explore the brain activity of BECTS patients in different frequency bands. Magnetoencephalography (MEG) is a non-invasive technique for measuring the neuromagnetic activity and locating the source of the neuromagnetic signals (Xiang et al., 2013). Moreover, MEG has a higher sampling rate than fMRI.

Our study aimed to investigate the characterization of BECTS specific neuromagnetic activities in multiple-frequency bands compared to healthy subjects. Due to transient and chronic abnormal activities in BECTS (Adebimpe et al., 2015; Zhu et al., 2015), we performed our study based on the presence and absence of IEDs to deepen the understanding of the pathogenesis of BECTS. And we attempted to use these differences in brain activity to distinguish BECTS without IEDs from healthy controls (HC). Considering that different frequency oscillations may have discrepant neurophysiological mechanisms and clinical significance(Lin et al., 2006; Tang et al., 2016), this study quantitatively analyzed the underlying relationship between neuromagnetic characteristics and clinical features from low- to -high-frequency bands in BECTS patients. In the present study, we hypothesized that the resting-state brain activities were significantly different between BECTS patients without IEDs and controls, particularly in the areas associated with cognitive function (Li et al., 2018) and oscillations were sensitive to specific frequency bands (Tan et al., 2018).

## Experimental Procedures

### Participants

Twenty-one children diagnosed with BECTS (age: 4.75–10.33 years) and 11 controls (age: 6.00–10.70 years) were recruited from the Department of Neurology at Nanjing Brain Hospital and Nanjing Children’s Hospital between March 2018 and July 2019. Written informed consent was achieved from children and their parents. The demographic and clinical details of the patients are shown in Table 1. The inclusion criteria for patients were as follows: a clinical and EEG diagnosis of BECTS according to the International League Against Epilepsy classification (2017) and normal cranial magnetic resonance imaging (MRI) results. The exclusion criteria were: the presence of other types of seizures, other neurological or mental disorders, parent-reported learning disabilities, or inability to hold their head still during MEG recording.

**Table 1:** Clinical characteristics of benign epilepsy with centrotemporal spikes (BECTS) patients.

Patients	Onset age (Years)	Duration (months)	Antiepileptic drugs	IEDs during MEG (Y/N)
1	8.00	16	No	Y
2	9.58	1	No	Y
3	9.50	1	No	Y
4	6.25	18	VPA	Y
5	4.67	1	No	N
6	8.92	7	LEV	N
7	8.17	5	No	Y
8	7.42	1	LEV	N
9	8.42	10	No	N
10	10.25	1	No	N
11	6.17	12	No	N
12	8.50	2	LEV	Y
13	7.08	12	OXC	Y
14	6.08	12	No	Y
15	6.67	2	LEV	Y
16	9.08	1	No	N
17	7.33	8	No	Y
18	6.75	11	No	N
19	8.67	7	No	N
20	4.92	14	VPA	Y
21	9.42	6	No	N

*VPA, valproic acid; LEV, levetiracetam; OXC, oxcarbazepine*.

### MEG Recording

The MEG signals were recorded in a magnetic-shielded room with a whole-head, 275-channel MEG system (VSM MedTech Systems, Inc., Coquitlam, BC, Canada) in the MEG Center of Nanjing Brain Hospital. All participants were required to reduce sleep time before MEG examination to increase the appearance of IEDs. To measure the participants’ head positions relative to the MEG sensors, three small coils were placed in each person’s left and right pre-auricular and nasion points before data acquisition. During recording, all subjects were asked to lie in a supine position comfortably, keep quiet, and stay still with their eyes closed but not to fall asleep (avoid swallowing or teeth clenching). For each control and patient, at least five consecutive epochs with a duration of 2 min were recorded. The sampling rate was 6,000 Hz.

### MRI Scan

All subjects underwent a 3.0T MRI (Siemens, Germany) scanning after MEG recording. To accurately co-register the MRI and MEG data, three fiduciary marks were put in the same locations of the three coils used in MEG recording. All anatomical landmarks digitized in the MEG recording were made identifiable in the MRI.

### Data Preprocessing

**Figure 1:**
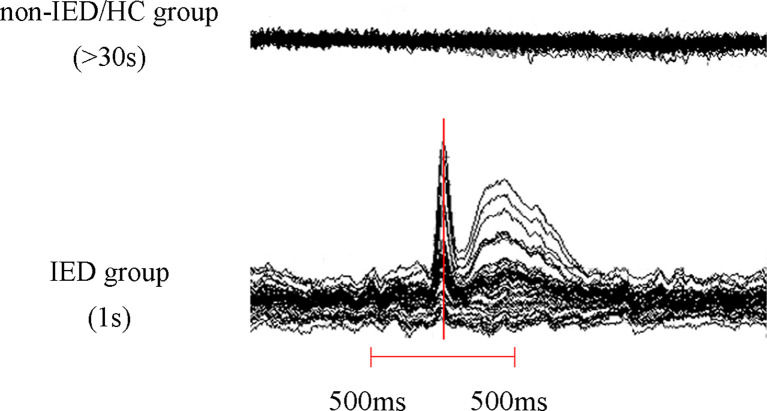
Schematic of magnetoencephalography (MEG) data analysis. At least 30 s of MEG data was recorded in the non-interictal epileptiform discharges (IED) group and the healthy controls (HC) group and a 1 s segment (500 ms before and 500 ms after the maximum negative peak of the spike) MEG data was selected in the IED group.

We visually examined MEG waveforms to exclude magnetic noise and artifacts (deflection of the MEG waveform >6 pT) before data analysis. The IEDs were labeled separately by two experienced magnetoencephalographers according to both spatial distribution and morphology. The patients were divided into two groups, the IED and non-IED groups, based on whether there were IEDs during the MEG. For the IED group, given the active epileptic transient state of IEDs, data analysis was performed in a time window of 1 s, covering from 500 ms before to 500 ms after the peak of the most prominent negative peak of the sharp wave. For non-IED patients and HC, a duration of at least 30 s was measured as interictal segments need to be recorded over a long period to ensure data stability (Figure 1).

### Source Localization

We analyzed magnetic sources in seven frequency bands: 1–4 Hz (delta), 4–8 Hz (theta), 8–12 Hz (alpha), 12–30 Hz (beta), 30–80 Hz (gamma), 80–250 Hz (ripple) and 250–500 Hz (fast ripple). Fifty Hertz power-line noise was avoided by using notch filters. Similar to previous studies (Xiang et al., 2014; Wu et al., 2017), accumulated source imaging (ASI) was used to localize neuromagnetic sources. ASI was defined as the volumetric summation of source activity during a period, which was specifically developed and optimized to analyze epileptic activity in multiple frequency ranges. Such algorithms localize correlated sources using node-beam lead fields. Since each node-beam lead field represents a form of “source-beamformer” or “sub-space solution,” ASI uses multiple source beamformers to separate correlated sources. The mathematical algorithms were performed by a software package named MEG Processor, which also measured neuromagnetic source strength (Xiang et al., 2014). ASI can be described by the following equation:

$$\text{Asi}(r,s)=\sum_{t=1}^{t=n}Q(r,t)$$

In Equation, Asi indicates the accumulated source strength at location *r*; *s* indicates the time slice; *t* indicates the time point of the MEG data; *n* indicates the total time points of the MEG data and *Q* indicates the source activity at source *r* and at time point *t*. We defined s ≥1 and *s* ≤*n*/2. We used two-step beamforming to calculate the source activity. Specifically, the first step was to compute lead fields for each source (or voxel position) to generate matrices with MEG data. The second step was to select sensors for partial sensor coverage for each voxel with the main field (Xiang et al., 2015b), called voxel-based partial sensors. In the following beamformers, voxel-based partial sensors were used to minimize the impact of coherent sources in source localization. Once the covariance for voxel-based partial sensors was computed, the third step was to compute two sets of magnetic source images using a vector beamformer (Xiang et al., 2015b). The next step was to estimate coherent source and source direction by using the covariance matrix-vector beamformer. After the source orientation was determined, the final step was to generate the source activity (or virtual sensor waveform) through a scalar beamformer (Xiang et al., 2015a). Previously published articles (Xiang et al., 2014, 2015b) have explained the detailed mathematical algorithms and validations.

The whole brain was scanned at a resolution of 6 mm (approximately 17,160 voxels/sources). If the distance between two voxels was less than 10 mm, they were considered as one source. To segment brain regions, individual MRI was incorporated into the MEG source imaging by using three fiducial points: the left and right pre-auricular and the nasion points. Anatomical cortical brain regions were defined by cerebral landmarks including the central sulcus, Sylvian fissure, and the somatosensory cortex. By combining MEG sources with individual MRI, we could visualize and segment brain regions in both 2D and 3D environments.

### Statistical Analyses

Fisher’s exact test was performed on predominant neuromagnetic source locations and the significance level was set at *p* < 0.05. Bonferroni multiple comparisons correction was used for pairwise comparison of the three groups (e.g., for three groups, *p* < 0.0167). One-way analysis of variance (ANOVA) was performed on the source strength and a homogeneity test of variance was carried out. A Bonferroni test was used for homogeneity of variance and the Tamhane T2 test was used for inhomogeneity of variance. Pearson’s correlation was used to analyze the correlation between the clinical characteristics and the strength of each source for each frequency band. The threshold of statistical significance was set at *p* < 0.05. All Statistical analyses were performed with SPSS version 24.0 for Windows (SPSS Inc., Chicago, IL, USA).

## Results

### Source Location

**Figure 2:**
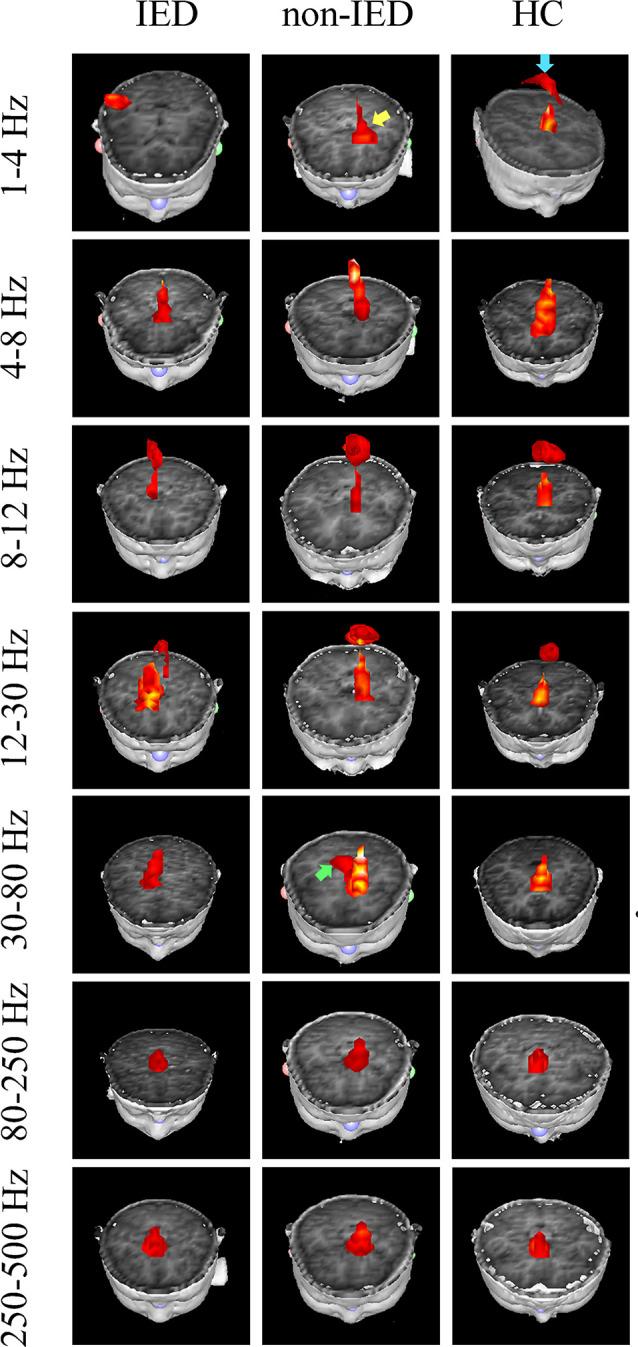
Magnetic source images showing intrinsic brain activity in a 1–500 Hz frequency range in three groups. The yellow arrow points to the region which shows a significant difference between the benign epilepsy with centrotemporal spikes (BECTS) patient with and without IEDs. Blue arrow points to the region that shows the activation in the control but not in the patient with IEDs. The green arrow indicates the predominant neuromagnetic activity between the BECTS without IEDs and the control.

The whole-brain magnetic source imaging (Figure 2) revealed that each subject typically had 2–3 sources stronger than the rest of the brain activity and the magnetic sources with the higher strength were mainly located in the peri-Rolandic area (PR), precuneus/posterior cingulate (PC/PCC), medial frontal cortex (MFC) and medial temporal lobe (MTL). Significant differences in the resting-state brain activities between the three groups at low-frequency bands (<80 Hz) could be visually identified. No significant differences were found at high-frequency bands.

At the delta band (1–4 Hz), the neuromagnetic activities of the three groups were different in the PR, PC/PCC, and MFC. Further pairwise comparison noted that compared to the IED group, the HC group tended to locate at the PC/PCC and the non-IED group tended to locate at the MFC. At the beta band (12–30 Hz), three groups differed in the PR, but further pairwise comparison revealed no significant differences. At the gamma band (30–80 Hz), the neuromagnetic source in the non-IED group showed high odds in the MTL compared to the HC group. Detailed statistical results for these source locations are shown in Table 2. Representative source images are shown in Figure 2.

**Table 2:** Predominant neuromagnetic activities in interictal epileptiform discharges (IED), non-IED and healthy controls (HC) group.

Frequency band (Hz)	Source location	Non-IED	IED	HC	*p*-value	Non-IED vs. IED (*P*-value)	Non-IED vs. HC (*P*-value)	IED vs. HC (*P*-value)
1–4	PR	3	7	1	0.027*	0.198	0.311	0.024
	PC/PCC	1	0	7	0.001*	0.476	0.024	0.004**
	MFC	10	5	10	0.007*	0.012**	1.000	0.063
	MTL	0	0	0	1.000	–	–	–
4–8	PR	3	7	3	0.234	–	–	–
	PC/PCC	3	2	4	0.709	–	–	–
	MFC	9	9	11	0.512	–	–	–
	MTL	0	0	0	1.000	–	–	–
8–12	PR	0	2	0	0.313	–	–	–
	PC/PCC	4	2	6	0.207	–	–	–
	MFC	9	8	8	0.649	–	–	–
	MTL	0	0	0	1.000	–	–	–
12–30	PR	0	5	1	0.029*	0.035	1.000	0.149
	PC/PCC	1	2	6	0.085	–	–	–
	MFC	9	6	8	0.297	–	–	–
	MTL	0	0	0	1.000	–	–	–
30–80	PR	1	4	0	0.062	–	–	–
	PC/PCC	0	0	0	1.000	–	–	–
	MFC	9	10	8	0.579	–	–	–
	MTL	9	7	2	0.003*	0.311	0.002**	0.080
80–250	PR	0	0	0	1.000	–	–	–
	PC/PCC	0	0	0	1.000	–	–	–
	MFC	6	6	8	0.737	–	–	–
	MTL	9	7	7	0.336	–	–	–
250–500	PR	0	0	0	1.000	–	–	–
	PC/PCC	0	0	0	1.000	–	–	–
	MFC	9	6	9	0.202	–	–	–
	MTL	10	9	8	0.327	–	–	–

PR, peri-Rolandic area; PC/PCC, precuneus/posterior cingulate; MFC, medial frontal cortex; MTL, medial temporal lobe. *p < 0.05 after Fisher’s exact test; **p < 0.0167 after Bonferroni multiple.

### Source Strength

One-way ANOVA suggested that at all frequency bands, the source strength of the non-IED group was different from the IED group and patients with IEDs exhibited higher activity. In addition to the 30–80 Hz frequency band, the source strength was significantly different between the IED group and the HC group. Compared to the controls, the strength of brain activity in the 30–80 Hz frequency seemed to be weaker in the non-IED group. Detailed statistical analyses of the neuromagnetic strengths are shown in Table 3.

**Table 3:** Analysis of neuromagnetic source strength between IED, non-IED and HC group.

Frequency band (Hz)	Source strength			ANOVA (*F*-value)	Non-IED vs. IED (*P*-value)	Non-IED vs. HC (*P*-value)	IED vs. HC (*P*-value)
	Non-IED	IED	HC				
1–4	76.76 ± 5.20	120.25 ± 24.50	78.30 ± 10.55	25.937	0.000**	0.965	0.000**
4–8	82.61 ± 7.22	120.27 ± 24.27	82.42 ± 6.43	22.042	0.001*	1.000	0.001*
8–12	84.59 ± 10.60	131.05 ± 18.30	90.17 ± 13.05	33.089	0.000**	1.000	0.000**
12–30	71.79 ± 5.96	102.34 ± 13.97	72.51 ± 3.00	40.383	0.000**	1.000	0.000**
30–80	49.21 ± 9.13	60.89 ± 7.26	57.26 ± 2.62	7.989	0.001*	0.034*	0.667
80–250	40.27 ± 10.19	54.79 ± 3.53	41.41 ± 2.16	18.431	0.004*	0.982	0.000**
250–500	37.29 ± 1.81	44.34 ± 2.52	35.31 ± 1.50	62.003	0.000**	0.092	0.000**

*p < 0.05 after Bonferroni multiple comparisons; **p < 0.001 after Bonferroni multiple comparisons.

### Clinical Correlation

**Figure 3:**
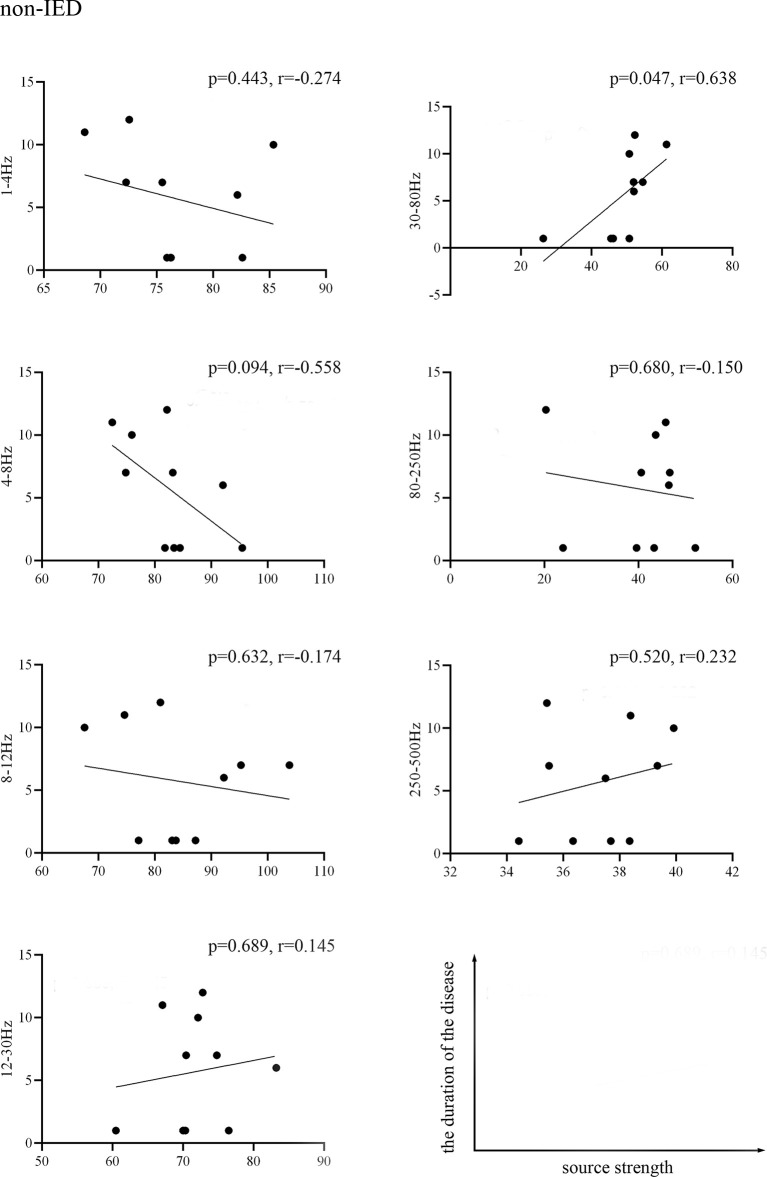
Pearson’s correlation showing the relationship between the source strength of the non-IED group activities and the duration of epilepsy. Source strength was found to be positively correlated with the duration of epilepsy in the 30–80 Hz band (*p* < 0.05, *r* = 0.638). Correlation between the two variables is plotted in the sample coordinate (last row) with source strength as abscissa and the duration of the disease as ordinate.

We found that the source strength in the 30–80 Hz gamma-band of the non-IED group was positively correlated with the duration of the disease (*p* = 0.047, *r* = 0.638). No significant correlations were detected within the IED group. Figure 3 shows the results of the correlations between the MEG measurements and the duration of the disease.

## Discussion

The current study demonstrated that during the interictal period, frequency-dependent neuromagnetic activities were different between the BECTS patients with and without IEDs, and between BECS without IEDs and controls. This was the first study to perform a multiple frequency analysis of source location in BECTS patients with and without IEDs by MEG.

Source analysis at 1–4 Hz revealed that neuromagnetic activity in the PC/PCC was decreased during transient abnormal discharge relative to control subjects. Meanwhile, at the delta band, we found that BECTS patients without IEDs had predominant activity in the MFC while the IED group did not. The PC/PCC and MFC are included in the brain regions of the default mode network (DMN), which is active in the resting state of the brain, but becomes deactivated when task performance is initiated (Mohan et al., 2016). The PC/PCC and MFC source depression in the IED are likely to be associated with decreased activity of the DMN (Archer et al., 2003; Gotman et al., 2005) and may contribute to reduced responsiveness during epileptic transients (Fahoum et al., 2013). Adebimpe et al. (2015) found alterations in the DMN in BECTS patients when they analyzed the influence of centrotemporal spikes on EEG spectral changes, and the abnormal brain region was found in the bilateral frontal areas. Besides, An EEG-fMRI study (Fahoum et al., 2013) suggested that deactivation occurred in various DMN regions, most frequently the precuneus and inferior parietal lobule, during IEDs. The reduced activity of the MFC might also explain some of the cognitive impairments and other brain malfunctions related to benign epilepsy (Holmes and Lenck-Santini, 2006), as it has been shown that any dysfunction in the frontal lobes in childhood is likely to affect cognitive development (Stuss and Alexander, 2000; Badre et al., 2009).

The analyses of neuromagnetic gamma activity (in the 30–80 Hz frequency range) suggested that the MTL was involved in patients without IEDs compared to healthy subjects. Diffusion tensor imaging studies confirmed that increased functional connectivity within the temporal and prefrontal regions supports increased the efficiency of memory processes, likely through a major white matter tract, the uncinate fasciculus (Mabbott et al., 2009; Verrotti et al., 2014). Based on functional neuroimaging studies, MTL structures are essential for long-term memory (Ofen et al., 2012; Ofen, 2012). Previous studies have identified the involvement of the MTL in BECTS (Jun et al., 2019) and white-matter abnormalities have been noted in the MTL (Lundberg et al., 1999). Additionally, a significant decrease in source strength in the 30–80 Hz frequency range was found in the non-IED group. The mechanism, however, is not clear. A MEG study (Xiang et al., 2015b) reported enhanced source activities in children with epilepsy compared to that in HC. Nevertheless, several studies have shown reduced brain activity resulting from the use of antiepileptic drugs (Béla et al., 2007; Clemens et al., 2007; Jun et al., 2019). These results demonstrated that even in the absence of IEDs, chronic changes in physiological neuronal activity occur in BECTS. This finding agrees with those reported in other resting-state studies (Pardoe et al., 2013; Adebimpe et al., 2015) and, collectively the data suggest that gamma frequency band could be used to distinguish BECTS without IEDs from control subjects. Gamma oscillations have been recognized to play an important role in various cognitive functions, including perception, attention, and working memory (Sohal, 2012; Kucewicz et al., 2017). Abnormal expression of gamma oscillations exists in different neurological and psychiatric disorders (Corlier et al., 2016).

In our study, the positive correlation between duration and source strength at gamma-band may indicate that gamma activity could assess the course of BECTS patients without IEDs. The neural oscillations in different brain regions are sensitive to specific frequency realm. Even within the same brain region, oscillations in different frequency bands have distinct physiological relevance (Tan et al., 2018). Our finding further confirmed the frequency-dependent correlation between neuromagnetic activity and some clinical features (Tang et al., 2016; Frauscher et al., 2017; Tan et al., 2018).

Furthermore, this study suggested that low-frequency and high-frequency activities were located in different brain regions and that high-frequency oscillations were involved in regions of the inner brain. Although the mechanism is not clear, studies have shown that different frequencies may facilitate different types of connections and/or integration of different information (Lopes da Silva et al., 2003; Xiang et al., 2015b).

Several limitations of the present study should be acknowledged. First, we need a larger sample size to improve the statistical power and generalize the results. Second, artifacts from electromyography, magnetocardiography, and other signals might interfere with MEG scanning, although we have tried to minimize the artifacts. Specifically, MEG signals have been recorded under the same experimental conditions, and we analyzed MEG data at the source level using accumulated technology. Third, the antiepileptic medications taken by some patients may have confounded the resting-state brain activity results. Lastly, we didn’t perform a synchronous EEG recording during the MEG scan. The lack of EEG data might contribute to the miss-reading of the MEG spikes. So two experienced magnetoencephalographers separately labeled the IEDs according to both spatial distribution and morphology.

## Data Availability Statement

All datasets generated for this study are included in the article.

## Ethics Statement

The studies involving human participants were reviewed and approved by Nanjing Brain Hospital Affiliated to Nanjing Medical University. Written informed consent to participate in this study was provided by the participants’ legal guardian/next of kin.

## Author Contributions

TZ, QS, and YL designed the study. YG, JS, CW, and ZH acquired the data, while YL, QC, and HG analyzed the data together. TZ and QS wrote the manuscript, while AM and XW revised it. All authors signed the final approval for publication.

## Conflict of Interest

The authors declare that the research was conducted in the absence of any commercial or financial relationships that could be construed as a potential conflict of interest.
